# A medical epopee: recurrent fungal endocarditis, heart transplantation and chylopericardium

**DOI:** 10.1186/s12872-020-01755-z

**Published:** 2020-10-31

**Authors:** Antonio de Santis, Guilherme Moratti Gilberto, Sandrigo Mangini, Adalberto Batalha Megale, Fabio Antonio Gaiotto, Ricardo Mingarini Terra, Rodrigo Gobbo Garcia

**Affiliations:** 1 Department of Cardiology, Hospital Israelita Albert Einstein, Albert Einstein Av. 627, São Paulo, Brazil; 2 Deparment of Interventional Radiology, Hospital Israelita Albert Einstein, São Paulo, Brazil; 3 Department of Cardiothoracic Surgery, Hospital Israelita Albert Einstein, São Paulo, Brazil; 4 Heart Institute (InCor), University of São Paulo Medical School, Dr Eneas de Carvalho Aguiar Street 44, São Paulo, Brazil

**Keywords:** Infective endocarditis, Heart transplantation, Pericardium

## Abstract

**Background:**

Candida prosthetic endocarditis is associated with high mortality rates and valve replacement surgery, together with antifungal treatment, play a major role in eradicating the fungal infection. Valve reoperations in these scenarios may be relatively common due to the high infection relapse rates and, in some cases, heart transplantation may be an imposing therapy for infection resolution and for the heart failure related to the myocardial reoperation injury. Among the many postoperative complications related to heart transplantation, chylopericardium is a rare but challenging example.

**Case presentation:**

We report the case of a 55-year-old man who was admitted to our hospital with a 1-month history of progressive dyspnea and fatigue. His past medical history included four open-heart surgeries for aortic and mitral valve replacement due to recurrent *Candida parapsilosis* infective endocarditis. Transthoracic echocardiogram showed a markedly reduced left ventricular systolic function and normofunctioning bioprosthetic valves. An inotropic dependency condition led to heart transplantation surgery. In the early postoperative period, a persistent chylous fluid started to drain from the pericardial tube, compatible with the diagnosis of chylopericardium. The lack of clinical response to total parenteral nutrition and intravenous infusion of octreotide imposed the need of interventional radiology with diagnostic lymphography through cisterna chyli puncture and thoracic duct catheterization, confirming the presence of a lymphatic fistula. A successful treatment outcome was achieved with percutaneous thoracic duct embolization using coils and n-butyl-cyanoacrilate glue, possibiliting hospital discharge.

**Conclusions:**

Fungal endocarditis requires combined treatment (surgical and antimicrobial) for eradication. Valve replacement, while necessary, may lead to severe ventricular deterioration and heart transplantation may be the only viable therapeutic solution. Among the several postoperative complications of heart transplantation, chylopericardium is an uncommon and defiant example. Advances in interventional radiology like the percutaneous embolization allow a less invasive and highly efficient approach for this complication.

## Background

Some patients, like a Greek epic legend, face true medical challenges in real life. Candida prosthetic endocarditis represents a major defiance in clinical practice due to its devastating potential, requiring surgical intervention and prolonged antimicrobial therapy. Although rare, corresponding to less than 1% of all infective endocarditis, this condition determines high mortality and relapse rates [1]. In this scenario, the necessary cardiac reoperations could be associated with left ventricular function impairment and severe systolic heart failure, sometimes imposing the need of a more definitive therapeutic approach like heart transplantation [2]. Among the many complications related to cardiac transplantation, chylopericardium represents a rare but challenging example, adding another contest in the postoperative period. We present a unique case report that brought together this entire sequence of events.

## Case presentation

We present a clinical report of a 55-year-old male patient with a past medical history of four open-heart surgeries for aortic and mitral valve replacement. His first cardiac surgery was performed in 2009 and consisted in mitral valve replacement using a bioprosthesis due to symptomatic severe mitral regurgitation related to an anterior leaflet prolapse [3]. Due to regular sports practice and fearing major bleedings related to anticoagulant therapy the patient refused to receive a mechanical prosthesis at this point.

**Fig. 1:**
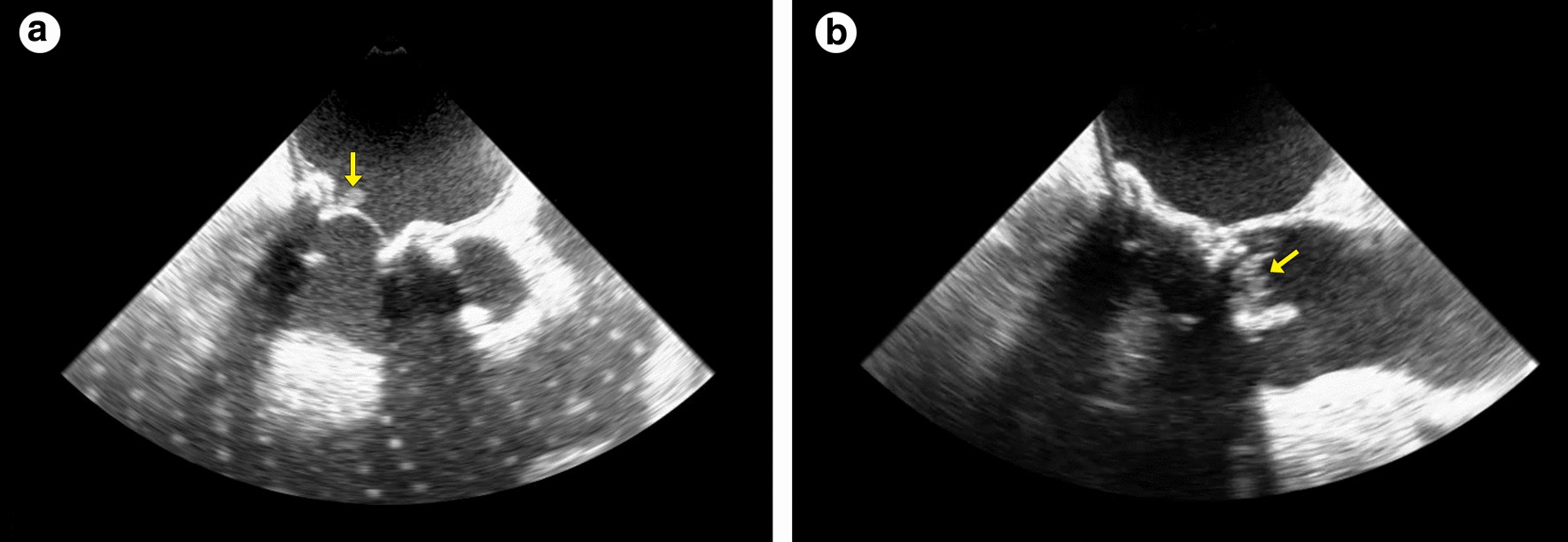
**a** A large fungal vegetation on mitral prosthesis (yellow arrow). **b** Aortic prosthesis vegetations (yellow arrow)

**Fig. 2:**
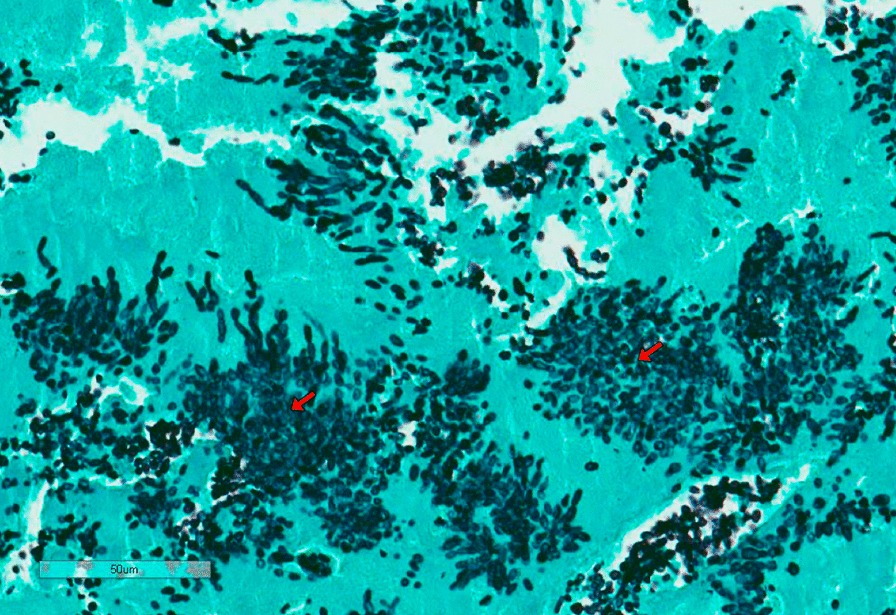
Histopathology of excised mitral bioprosthesis. Grocott’s methenamine silver stain showing fungal hyphae and yeasts (red arrows)

A year later, he received biological mitral prosthesis replacement for symptomatic severe paraprosthetic leak, with a good postoperative outcome [3]. In March 2013, he presented to the emergency department with a 2-week history of daily fever. Serial blood cultures were positive for *Candida parapsilosis* and transesophageal echocardiography showed multiple vegetations attached to the mitral prosthesis and native aortic valve. Given the diagnosis of fungal infective endocarditis, another cardiac surgical approach was necessary, consisting of combined mitral and aortic valve replacement using biological prostheses [4]. After a 6-week antifungal treatment with liposomal amphotericin-B, the patient was discharged from hospital on suppressive antifungal therapy with oral fluconazole for a year. After 3 years, the patient searched for emergency medical care with a 1-week history of high fever (39 °C). Once again, *Candida parapsilosis* was isolated from blood cultures and intravenous antifungal treatment with liposomal amphotericin-B was started. Candida endocarditis relapse was confirmed by transesophageal echocardiography, revealing mitral and aortic prosthesis with leaflet thickening and large vegetations with extension into the left atrium (Fig. 1, panels a, b). Then, the patient underwent his fourth operation: mitral and aortic prosthesis replacement (Fig. 2) [4]. Transient inotrope dependence resulted in a prolonged postoperative intensive care unit period, related to left ventricular dysfunction (ejection fraction of 40%). Once again, a new 6-week liposomal amphotericin-B cycle was performed. This time, the patient definitely remained on suppressive antifungal therapy with oral fluconazole.

During the follow-up period, there was a worsening of the left ventricular ejection fraction up to 25%. Hospitalizations for acute decompensated heart failure became recurrent despite optimized pharmacological treatment and cardiac resynchronization therapy.

Due to clinical deterioration he was admitted to the cardiology unit of our institution with acute decompensated heart failure, requiring inotropic support with dobutamine in addition to intravenous diuretic therapy. After multidisciplinary evaluation, he was included in the national heart transplantation waiting list with priority criteria (inotrope dependence) [5]. After 3 months of waiting, he finally underwent orthotopic heart transplantation. The initial postoperative period was marked by acute kidney injury, requiring continuous venovenous hemofiltration and hemodialysis. Fortunately, there was a favorable outcome, with effective weaning of vasoactive drugs and recovery of kidney function. Between the 10th and 12th postoperative days, a persistent massive chylous fluid, with a typical “milky” appearance, started to drain from the pericardial tube. The high triglyceride level in the aspirated pericardium fluid (800 mg/dl) was compatible with chylopericardium diagnosis. Initially, clinical treatment with total parenteral nutrition and intravenous infusion of octreotide was attempted. However, after 1 week of treatment, the maintenance of high chylous pericardial drainage configured a clinical therapeutic failure. After a muldisciplinary discussion, invasive lymphangiography was proposed.

**Fig. 3:**
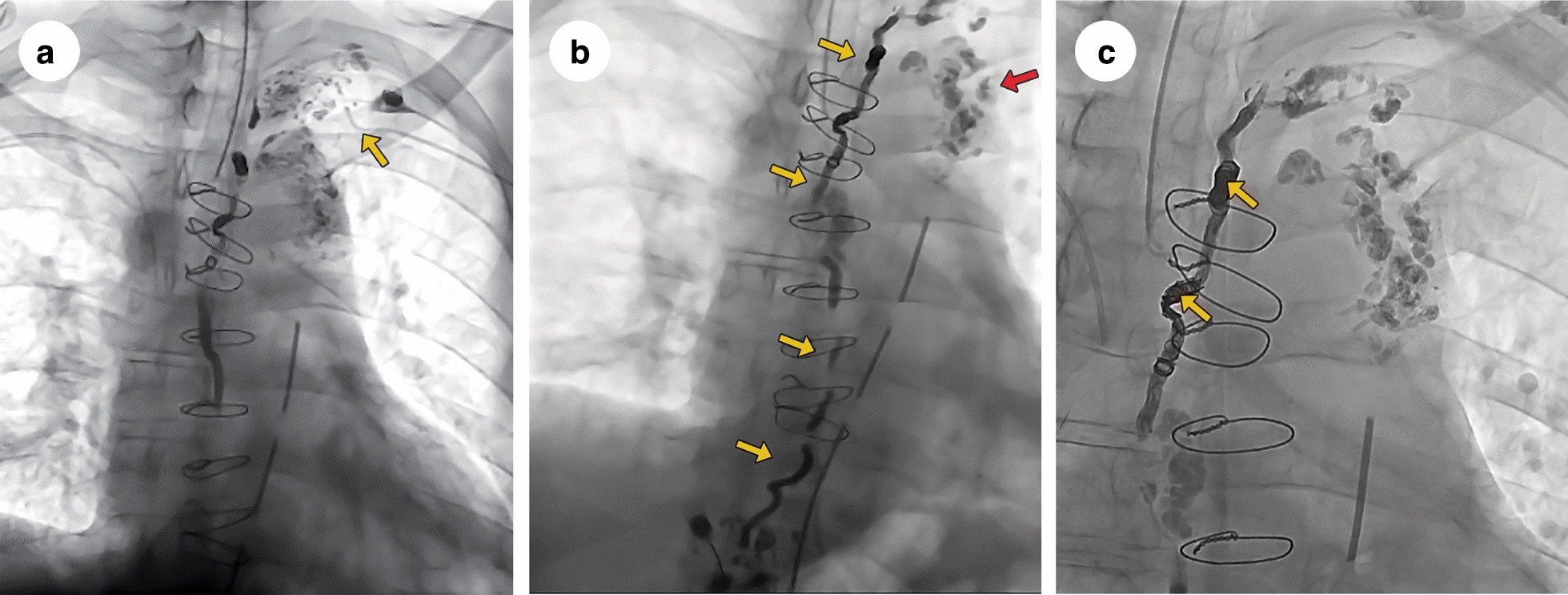
**a** Retrograde thoracic duct catheterization (yellow arrow) with lipiodol leakage into the pericardic sac. **b** Transhepatic cisterna chyli puncture. Note the lipiodol into the thoracic duct (yellow arrows) and the lymphopericardial fistula (red arrow). **c** Embolized thoracic duct with micro coils (yellow arrows)

Lymphangiography and thoracic duct embolization were performed in a dedicated interventional radiology suit, according to the following steps: (1) inguinal lymph nodes guided puncture followed by microbubble contrast infusion for regional lymphatic ducts anatomic evaluation; (2) lymphangiography with lipiodol infusion (Guerbet, Villepint, France); (3) the lack of contrast impregnation into the upper abdomen and cisterna chyli led to a retrograde access approach; (4) left upper limb phlebography allowed the thoracic duct ostium identification and its retrograde catheterization; (5) thoracic duct lymphangiography showed a lymphopericardial fistula with lipiodol leakage into the pericardical sac; (6) thoracic duct embolization using micro coils and n-butyl-cyanoacrilate glue was performed (Fig. 3, panel a). The amount of contrast (lipiodol) used was 50 ml and the duration of the procedure was 3 h.

In the first postoperative day, there was a significant reduction in the drainage flow (160 ml). However, in the subsequent days, there was a progressive increase in the daily drainage volume. A new approach was then performed by transhepatic percutaneous direct puncture of the cisterna chyli, using 50 ml of lipiodol contrast. Another thoracic duct embolization with liquid agent n-butyl-2-cyanoacrylate was achieved, taking 3 h of procedure (see Additional file 1 to watch the thoracic duct lymphangiography video confirming the lymphopericardial fistula). In the immediate postoperative period, there was a marked reduction in drainage flow, sustained in the subsequent days. (Fig. 3, panels b, c). Finally, after a prolonged period of hospitalization, the patient was discharged in excellent clinical conditions. Table 1 presents a timeline with the main reported events in this case (see Additional file 2 to access a slide set containing details of the case presentation). At the 6-months follow-up, the patient was in good clinical condition and pleased to have recovered his quality of life, without cardiovascular symptoms.

**Table 1:** Timeline of the main reported events

2009	2010	2013	2016	2019	2019 At the 10th to 12th postoperative day	2019 At the 20th and 24th posteoperative day	2019 At the 30th posteoperative day
Mitral valve replacement (bioprosthesis)	Mitral bioprosthesis replacement	Mitral and aortic valve replacement	Mitral and aortic bioprosthesis replacement	Heart transplantation	Chylopericardium	Thoracic duct percutaneous embolization	Hospital discharge
Indication: symptomatic anterior leaflet prolapse	Indication: Severe paraprosthetic leak	Indication: *Candida parapsilosis* endocarditis	Indication: *Candida parapsilosis* endocarditis recurrence	Indication: Severe systolic heart failure with inotrope dependence			

## Discussion and conclusions

### Recurrent fungal endocarditis and valve replacement procedures

Our patient experienced the dramatic natural history of candida prosthetic endocarditis, characterized by high morbidity mainly related to elevated recurrence rate (around 36%) despite combined treatment (valve replacement and long-term antifungal therapy) [1]. Candida prosthetic endocarditis is a rare entity, corresponding up to 3.4% of valvar prosthetic endocarditis, with a high mortality rate (up to 50% in a 5-year period) [3]. Interestingly, the patient did not have any risk factors related to fungal endocarditis such as immunosuppression, parenteral nutrition and intravenous drug use. In fact, the source of the fungal infection has not been identified in this case. From an etiological perspective, *Candida albicans* is the most frequently involved agent [4]. Despite valve replacement surgery, a 6-week in-hospital antifungal treatment with intravenous liposomal amphotericin-B and a year of continuous suppressive antifungal therapy with oral fluconazole the patient had prosthetic endocarditis recurrence 3-years later by the same agent: *Candida parapsilosis*. Concerning the factors related to this therapeutic failure and recurrence of the infection, it is possible that a longer period of postoperative suppressive antifungal therapy (> 1 year), as described in some case series, could prevent this relapse [3, 4].

After 4 open-heart cardiac surgeries, an inexorable complication arose for our patient: severe systolic left ventricular dysfunction. In a retrospective study, Ataka et al. found that up to 2/3 of patients with second/third valvar reoperation had a significant loss of ventricular function (around 25%), possibly related to ischemic/reperfusion injury in the previous operations [2]. Regarding this case, serial echocardiographic evaluation documented a progressive worsening of left ventricular function (up to 25%) associated with secondary pulmonary artery hypertension, without concomitant prosthetic valve dysfunction. Recurrent hospital admissions for decompensating heart failure requiring inotropic support have become frequent, despite pharmacological treatment optimization. Even the implementation of cardiac resynchronization therapy has not been able to mitigate symptoms and hospitalizations. In view of this poor evolution, the patient was referred for heart transplantation evaluation.

### Heart transplantation complicated by chylopericardium

Taylor et al. found that about 4% of patients undergoing heart transplantation had their cardiomyopathy related to a valvular pathology, representing a small proportion when compared to other etiologies such as ischemic or dilated cardiomyopathy [6]. The reasons for this low prevalence can be justified by the possibility of surgical correction (repair or valve replacement) in most of the valve diseases, with heart transplantation restricted to those subgroups considered inoperable or at high surgical risk. One of the major concerns related to heart transplantation for patients with valvular cardiomyopathy is the association with increased pulmonary vascular resistance, especially in mitral valve disease. This finding may impair the immediate postoperative period with right ventricular failure and hemodynamic deterioration.

After successful orthotopic heart transplantation, our patient was diagnosed with chylopericardium in the early postoperative period. Chylopericardium is a rare condition with variable clinical presentation, involving the development of a lymphatic fistula to the pericardium [7]. The most common etiologies are idiopathic (56%), after valve replacement surgery (9%), coronary artery bypass grafting (6%) and heart transplantation (3%) [7]. It is very likely that during the heart transplantation, the dissection of multiple adhesions related to previous redo operations has caused an accidental injury to the thoracic duct, culminating in the installation of the chylopericardium. Confirmatory testing requires a total triglycerides concentration at the pericardium effusion > 500 mg/dL, cholesterol/triglyceride ratio < 1.0, predominance of lymphocytic cells and negative cultures [7]. Conservative treatment may be successful in up to 50% of patients and recommended for patients with daily pericardium drainage less than 1000 mL. Low-fat adapted diets, rich in medium chain triglycerides, or even total parenteral nutrition are the main therapeutic strategies [7]. Surgical treatment usually consists of thoracic duct ligation [8]. However, the anatomical identification of the thoracic duct represents a major difficulty issue during surgery, leading to the development of less invasive therapies as percutaneous embolization [9]. The approach to the thoracic duct can be performed by anterograde access (via cisterna chyli puncture or direct thoracic duct catheterization) or by retrograde route through direct thoracic duct ostium catheterization into the subclavian vein [10, 11]. Embolization is usually performed by using liquid agents and/or micro coils with technical and clinical success rates up to 79% and 72%, respectively [12, 13]. In this case, our interventional radiology team selected the retrograde access for embolization. A second complementary embolization was performed three days later, via cisterna chyli direct puncture, with a successful fistula repair. A literature review identified four reported cases of chylopericardium after heart transplantation: in two cases, conservative treatment warranted clinical success; in the other cases, after an initial conservative treatment, surgical intervention was required for definitive resolution [12–15]. This is the first case report of thoracic duct embolization for chylopericardium treatment after heart transplantation. This technique can be effective and a less morbid alternative to open heart surgery.

Regarding the patient’s perspective, it is worth highlighting the strength that has always motivated him during the many intercurrences experienced. Despite some disappointments and moments of uncertainty, he has never lost confidence in the medical team. His willingness and cooperation were crucial for the positive outcomes achieved.

From an educational point of view, this case report brings some relevant conclusions: (1) fungal endocarditis requires a strict clinical follow-up after hospital discharge with special care in implementing and maintaining antifungal suppression therapy to avoid recurrence; (2) cardiac reoperations, even when necessary, can lead to permanent ventricular dysfunction; (3) heart transplantation remains a method for definitive clinical recovery in refractory heart failure; (4) integrated multidisciplinary work in the form of heart team is essential to ensure favorable clinical outcomes in such challenging scenarios.

## Supplementary information

**Additional file 1**. Thoracic duct lymphangiography video confirming the lymphopericardial fistula after infusion of the lipiodol contrast.

**Additional file 2**. Slide set containing details of the case presentation.

## Data Availability

The datasets used in the case are available from the corresponding author upon reasonable request.
