# Supplementary material for: A medical epopee: recurrent fungal endocarditis, heart transplantation and chylopericardium
Source: BMC Cardiovasc Disord. 2020 Oct 31;20:469. doi: 10.1186/s12872-020-01755-z (PMC7603709; doi:10.1186/s12872-020-01755-z)
Supplement: Supplementary file 2 — Additional file 2. Slide set containing details of the case presentation. [file 12872_2020_1755_MOESM2_ESM.ppt]

## Slide 1
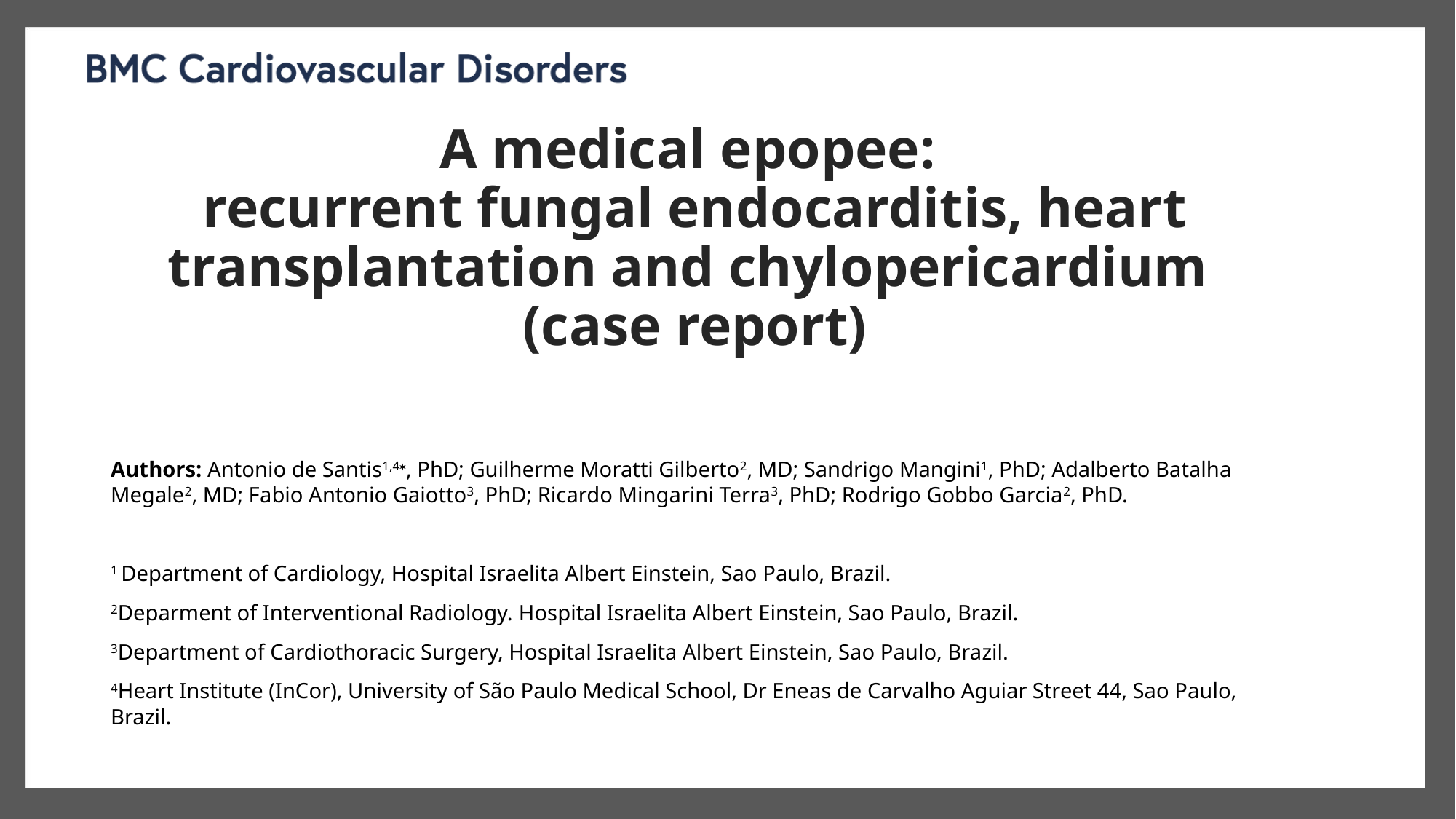

# A medical epopee: recurrent fungal endocarditis, heart transplantation and chylopericardium (case report)
Authors: Antonio de Santis1,4, PhD; Guilherme Moratti Gilberto2, MD; Sandrigo Mangini1, PhD; Adalberto Batalha Megale2, MD; Fabio Antonio Gaiotto3, PhD; Ricardo Mingarini Terra3, PhD; Rodrigo Gobbo Garcia2, PhD.
1 Department of Cardiology, Hospital Israelita Albert Einstein, Sao Paulo, Brazil.
2Deparment of Interventional Radiology. Hospital Israelita Albert Einstein, Sao Paulo, Brazil.
3Department of Cardiothoracic Surgery, Hospital Israelita Albert Einstein, Sao Paulo, Brazil.
4Heart Institute (InCor), University of São Paulo Medical School, Dr Eneas de Carvalho Aguiar Street 44, Sao Paulo, Brazil.

## Slide 2
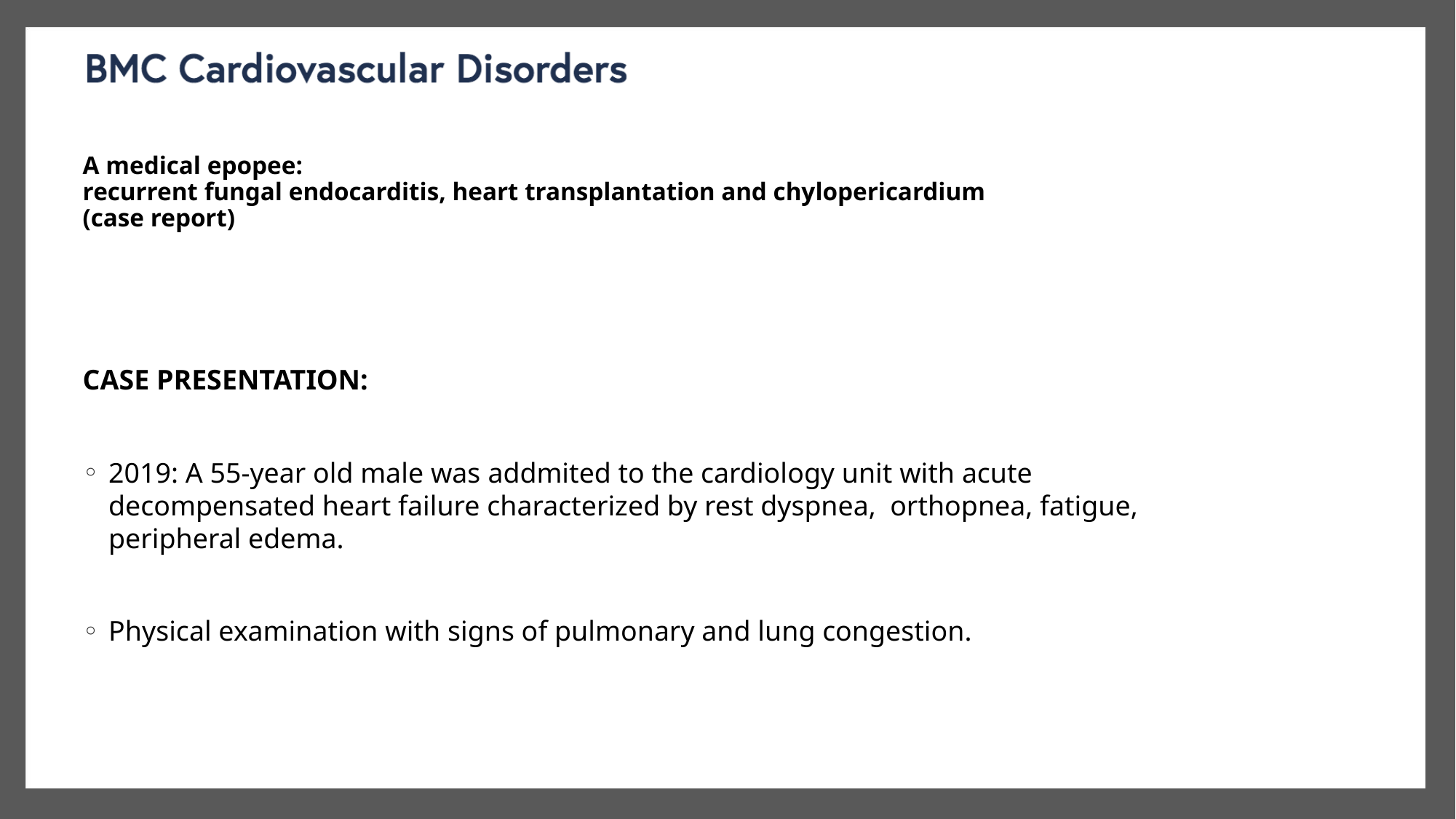

# A medical epopee: recurrent fungal endocarditis, heart transplantation and chylopericardium (case report)
CASE PRESENTATION:
2019: A 55-year old male was addmited to the cardiology unit with acute decompensated heart failure characterized by rest dyspnea, orthopnea, fatigue, peripheral edema.
Physical examination with signs of pulmonary and lung congestion.

## Slide 3
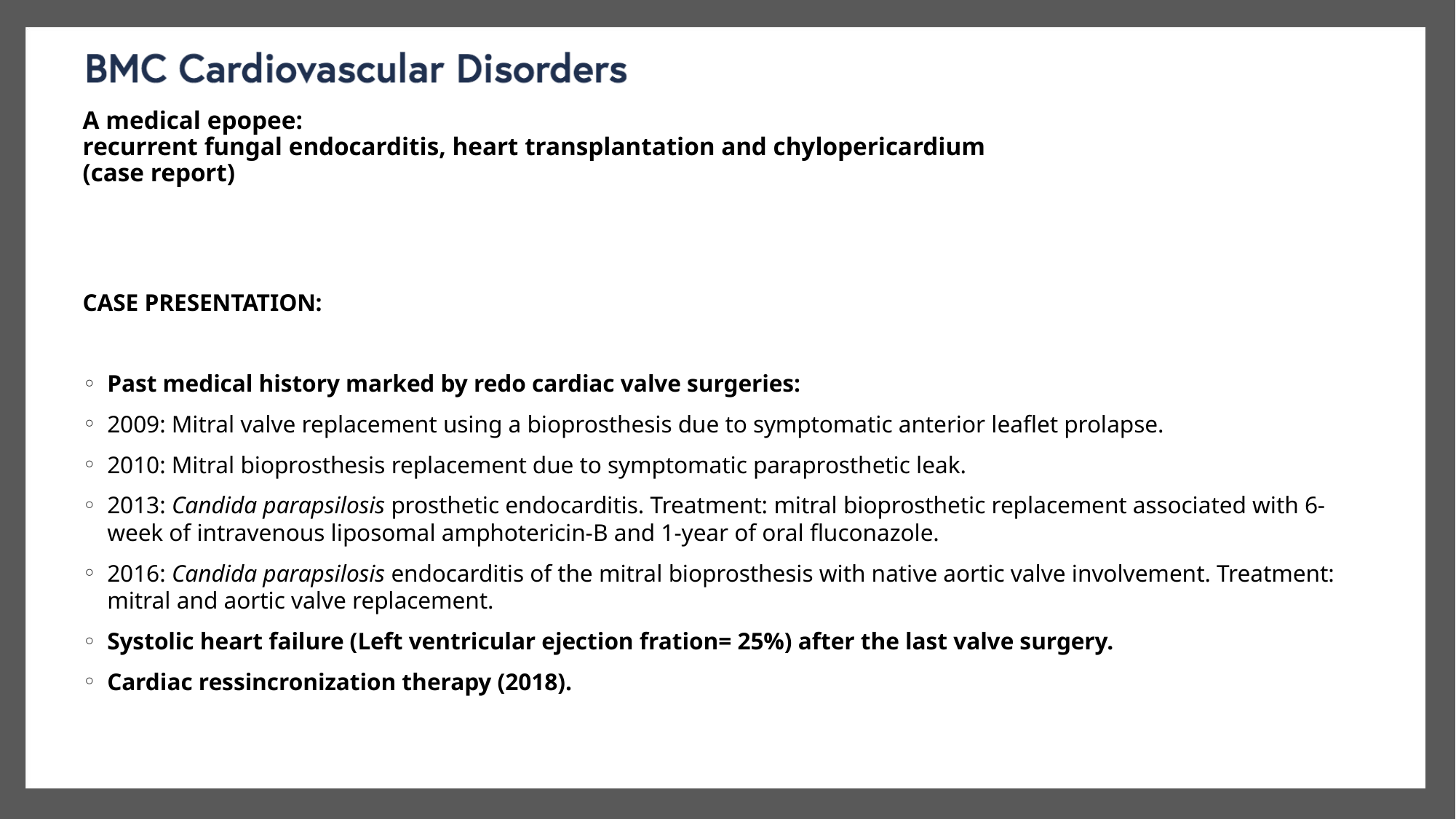

# A medical epopee: recurrent fungal endocarditis, heart transplantation and chylopericardium (case report)
CASE PRESENTATION:
Past medical history marked by redo cardiac valve surgeries:
2009: Mitral valve replacement using a bioprosthesis due to symptomatic anterior leaflet prolapse.
2010: Mitral bioprosthesis replacement due to symptomatic paraprosthetic leak.
2013: Candida parapsilosis prosthetic endocarditis. Treatment: mitral bioprosthetic replacement associated with 6-week of intravenous liposomal amphotericin-B and 1-year of oral fluconazole.
2016: Candida parapsilosis endocarditis of the mitral bioprosthesis with native aortic valve involvement. Treatment: mitral and aortic valve replacement.
Systolic heart failure (Left ventricular ejection fration= 25%) after the last valve surgery.
Cardiac ressincronization therapy (2018).

## Slide 4
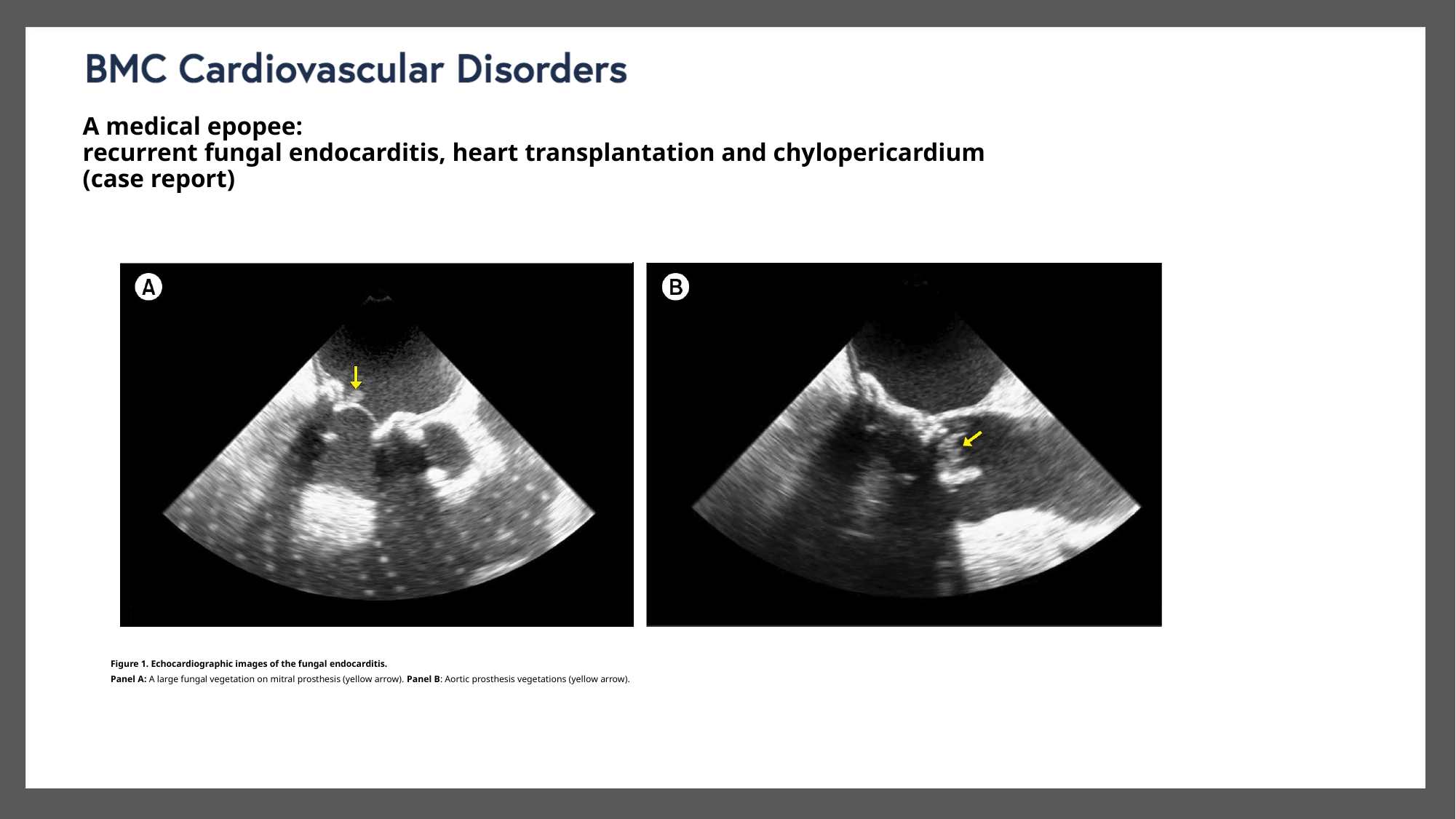

# A medical epopee: recurrent fungal endocarditis, heart transplantation and chylopericardium (case report)
Figure 1. Echocardiographic images of the fungal endocarditis.
Panel A: A large fungal vegetation on mitral prosthesis (yellow arrow). Panel B: Aortic prosthesis vegetations (yellow arrow).

## Slide 5
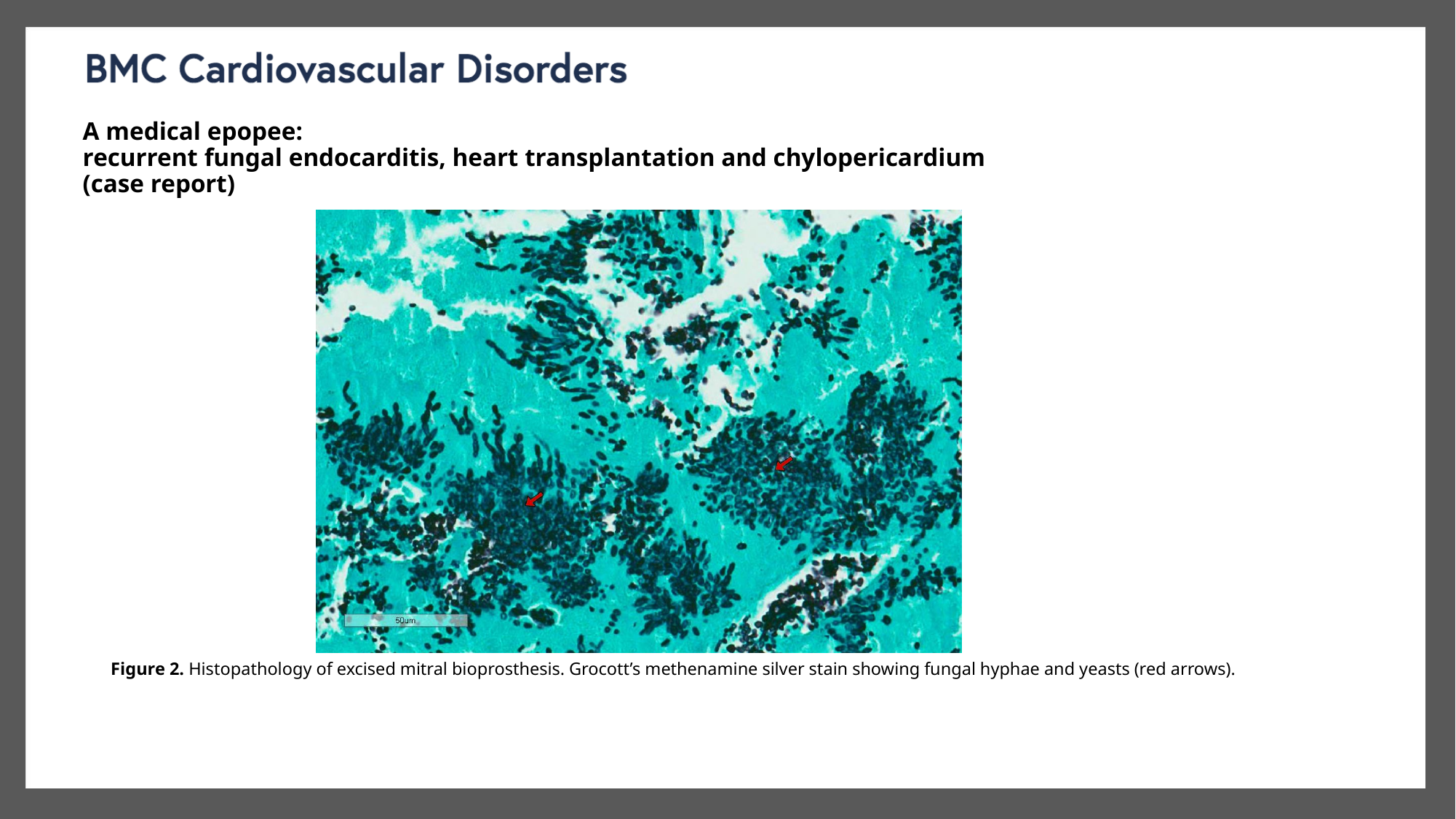

# A medical epopee: recurrent fungal endocarditis, heart transplantation and chylopericardium (case report)
Figure 2. Histopathology of excised mitral bioprosthesis. Grocott’s methenamine silver stain showing fungal hyphae and yeasts (red arrows).

## Slide 6
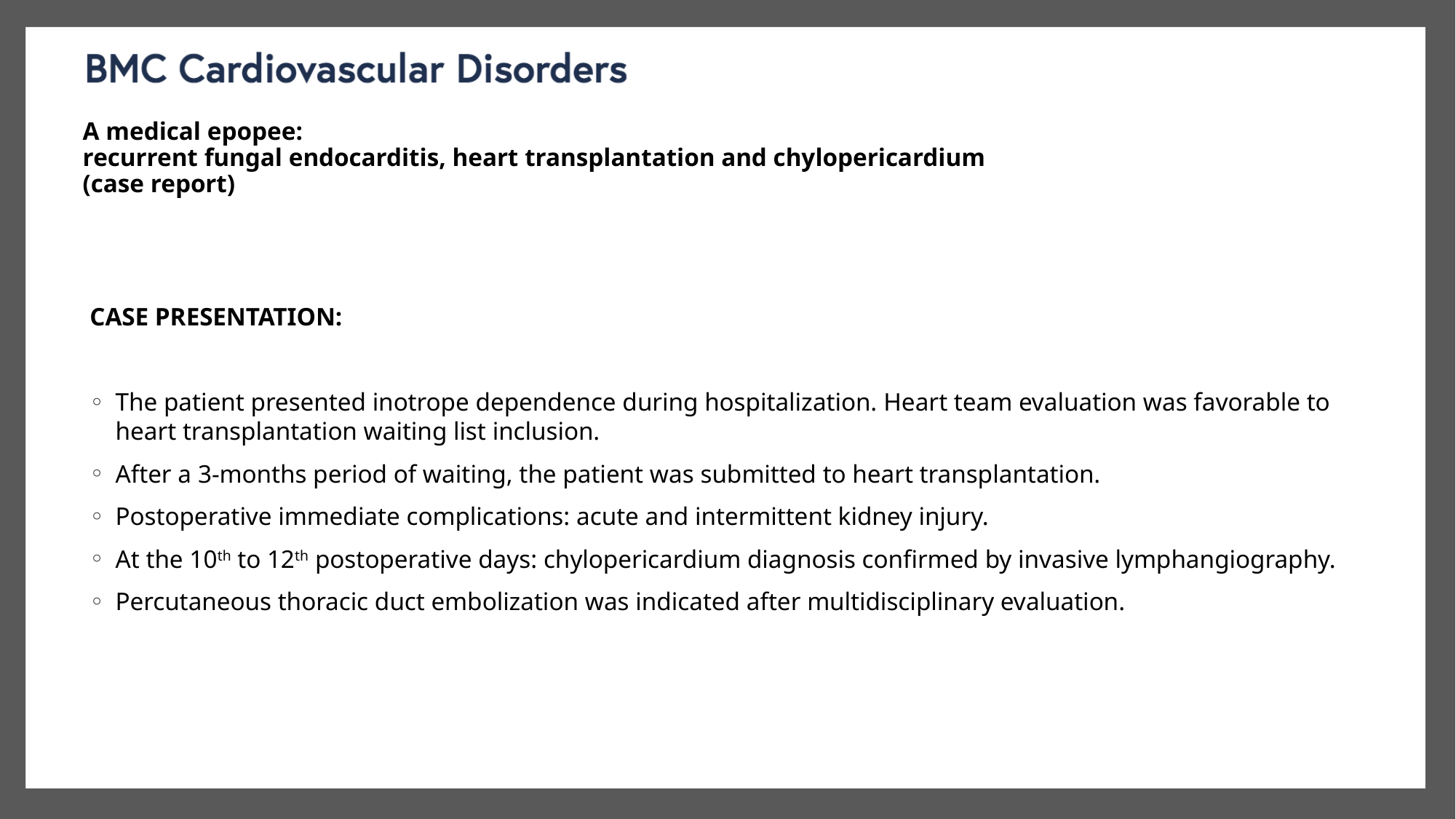

# A medical epopee: recurrent fungal endocarditis, heart transplantation and chylopericardium (case report)
CASE PRESENTATION:
The patient presented inotrope dependence during hospitalization. Heart team evaluation was favorable to heart transplantation waiting list inclusion.
After a 3-months period of waiting, the patient was submitted to heart transplantation.
Postoperative immediate complications: acute and intermittent kidney injury.
At the 10th to 12th postoperative days: chylopericardium diagnosis confirmed by invasive lymphangiography.
Percutaneous thoracic duct embolization was indicated after multidisciplinary evaluation.

## Slide 7
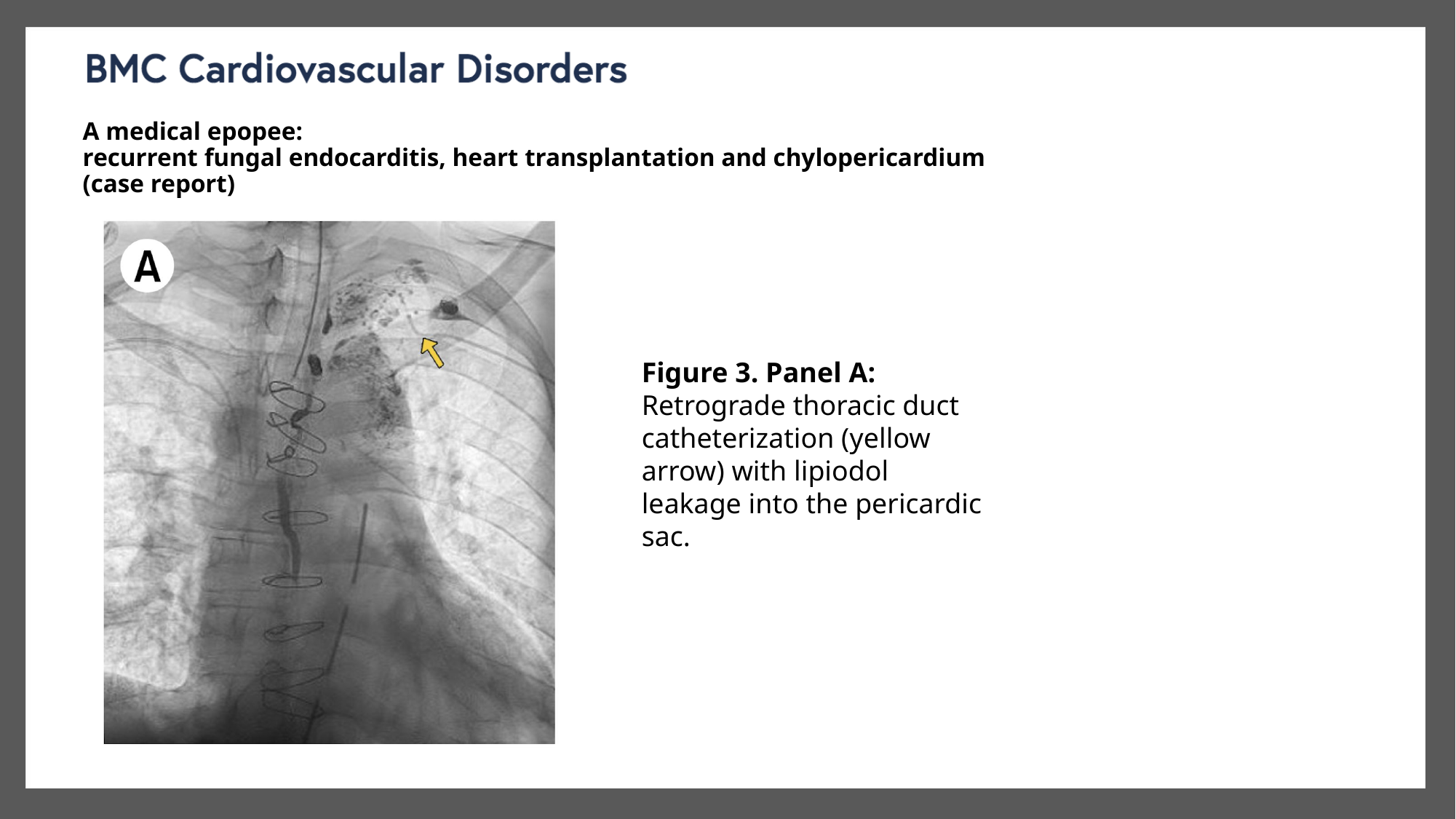

# A medical epopee: recurrent fungal endocarditis, heart transplantation and chylopericardium (case report)
Figure 3. Panel A: Retrograde thoracic duct catheterization (yellow arrow) with lipiodol leakage into the pericardic sac.

## Slide 8
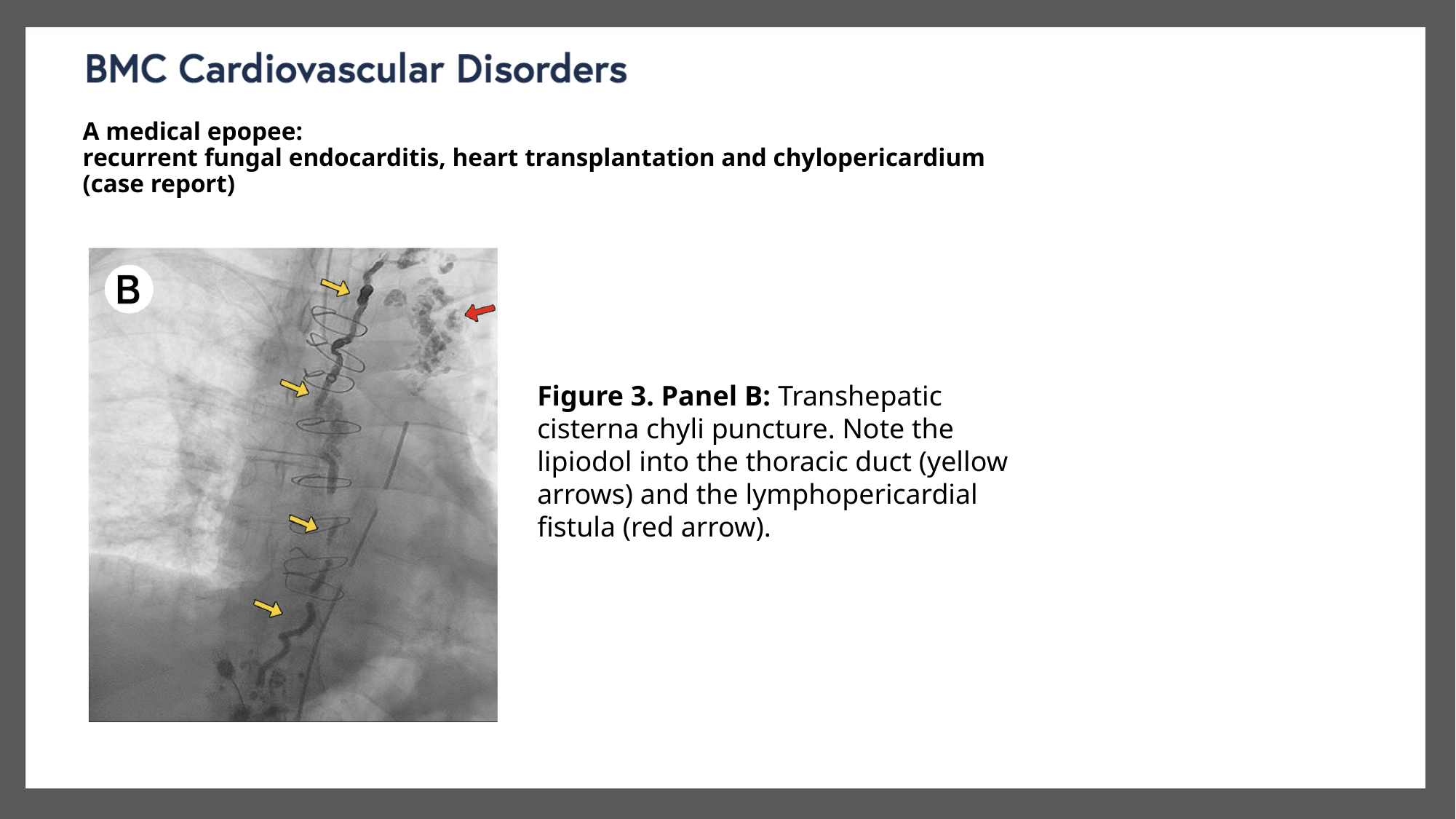

# A medical epopee: recurrent fungal endocarditis, heart transplantation and chylopericardium (case report)
Figure 3. Panel B: Transhepatic cisterna chyli puncture. Note the lipiodol into the thoracic duct (yellow arrows) and the lymphopericardial fistula (red arrow).

## Slide 9
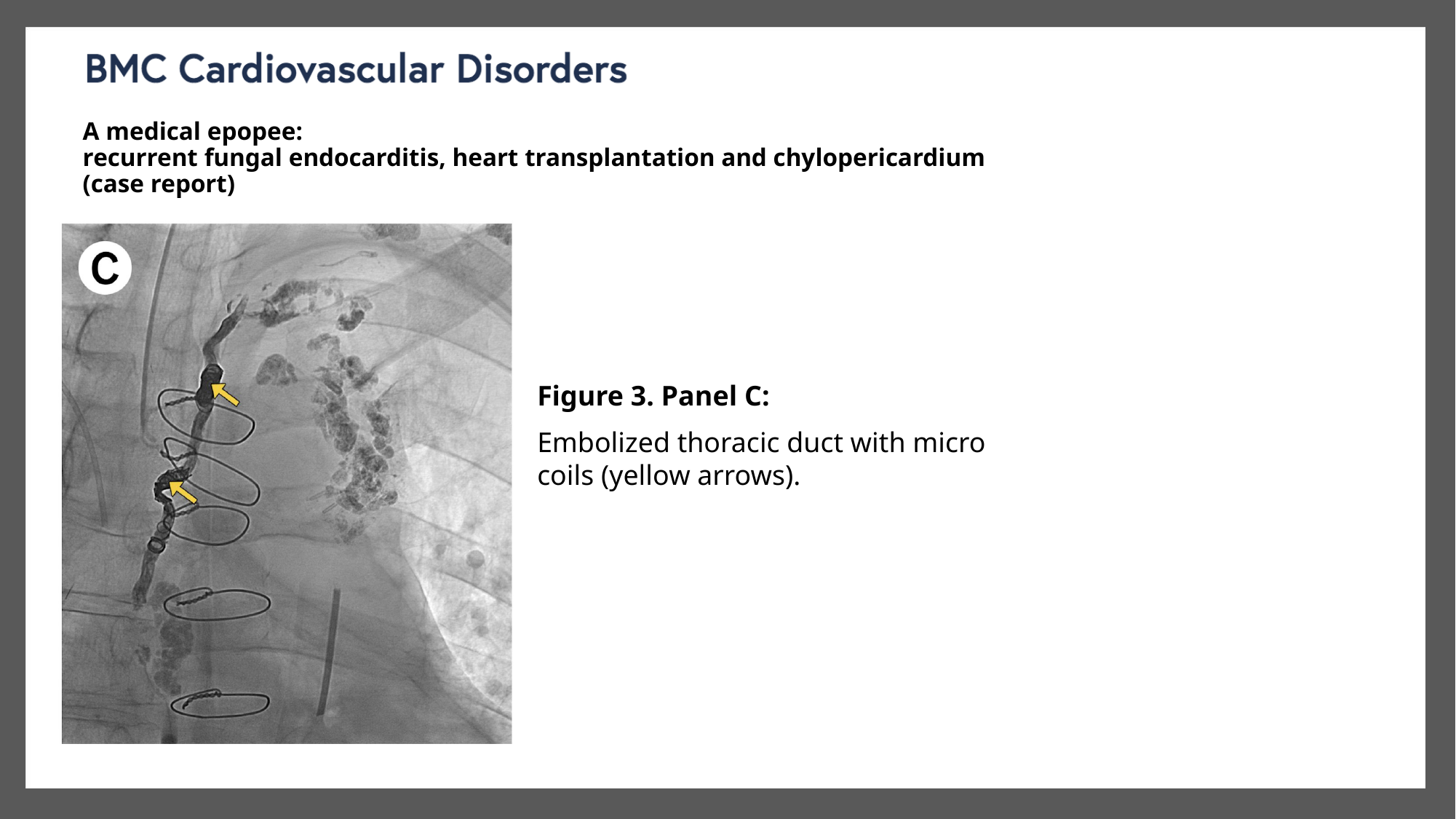

# A medical epopee: recurrent fungal endocarditis, heart transplantation and chylopericardium (case report)
Figure 3. Panel C:
Embolized thoracic duct with micro coils (yellow arrows).

## Slide 10
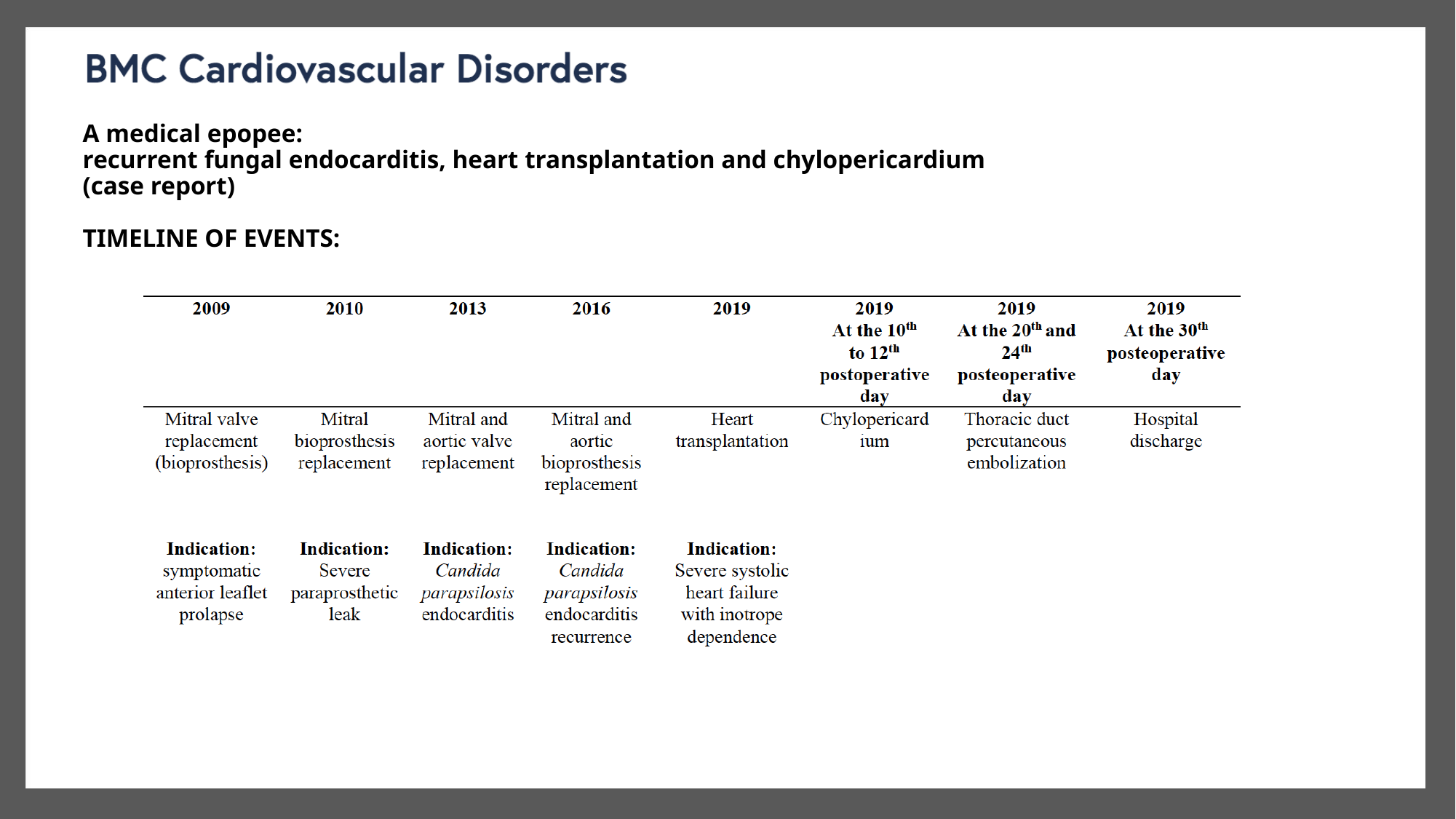

# A medical epopee: recurrent fungal endocarditis, heart transplantation and chylopericardium (case report) TIMELINE OF EVENTS:

## Slide 11
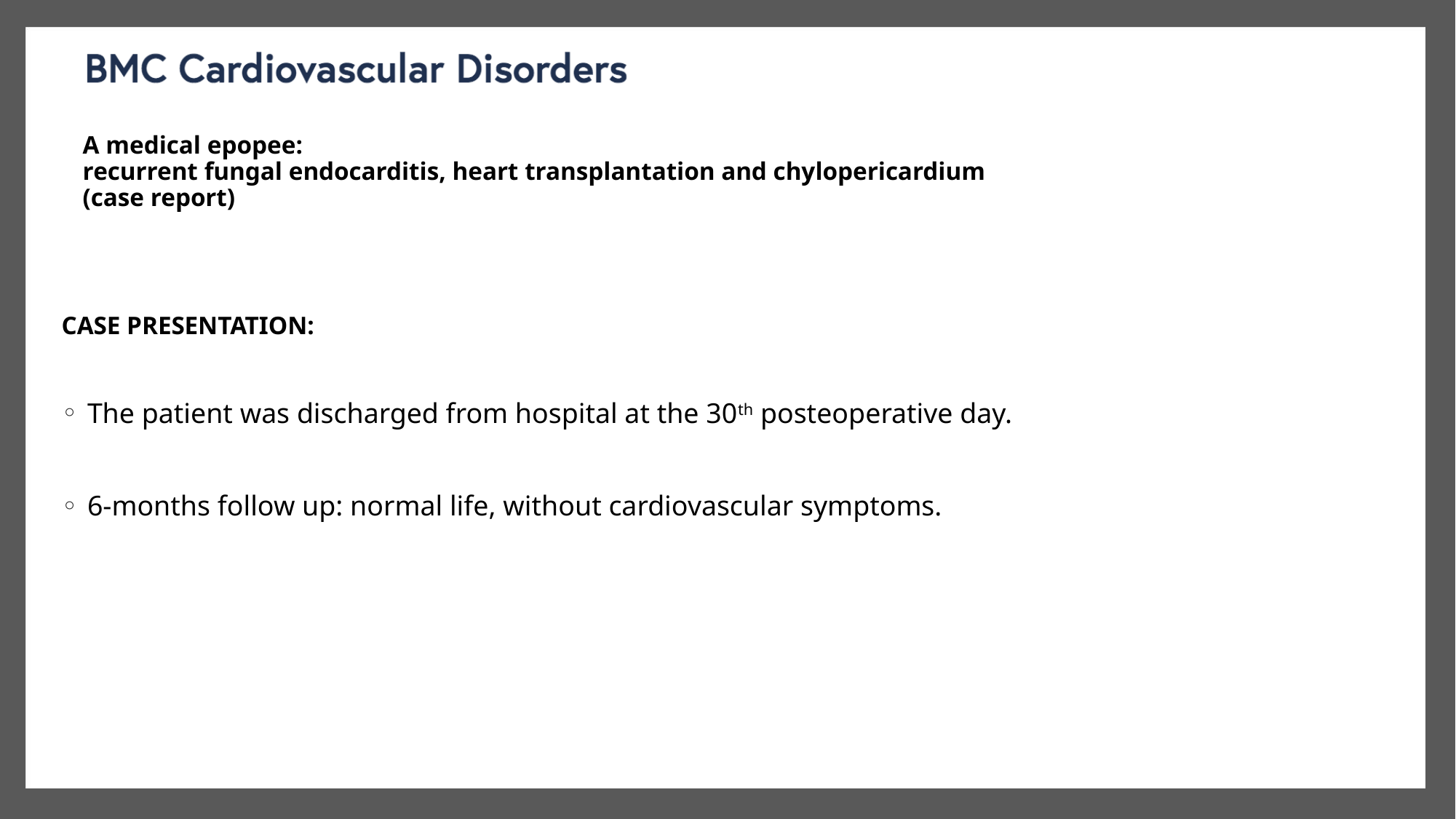

# A medical epopee: recurrent fungal endocarditis, heart transplantation and chylopericardium (case report)
CASE PRESENTATION:
The patient was discharged from hospital at the 30th posteoperative day.
6-months follow up: normal life, without cardiovascular symptoms.
